# Simultaneous endovascular revascularization of external iliac artery dissection and transplant renal artery stenosis in a recent renal allograft recipient

**DOI:** 10.1590/1677-5449.210035

**Published:** 2021-09-10

**Authors:** Rajesh Vijayvergiya, Atit Gawalkar, Ganesh Kasinadhuni, Ashish Sharma, Sarbpreet Singh, Anupam Lal

**Affiliations:** 1 Post Graduate Institute of Medical Education & Research – PGIMER, Chandigarh, India.

**Keywords:** endovascular stent, iliac artery dissection, renal artery angioplasty, renal transplantation, transplant renal artery stenosis, stent endovascular, dissecção da artéria ilíaca, angioplastia da artéria renal, transplante renal, estenose da artéria renal transplantada

## Abstract

Various vascular complications following renal transplantation include renal artery and vein thrombosis, renal artery stenosis, pseudoaneurysm, and iliac artery dissection. Transplant renal artery stenosis (TRAS) is the most common, while iliac artery dissection is the rarest of these various vascular complications. We describe an elderly male, who had both external iliac artery dissection and TRAS at 2 months following renal transplantation. He underwent successful percutaneous endovascular intervention of both complications. The post-intervention course was uneventful, with improvement in graft renal functions and left lower limb perfusion.

## Resumo

As diversas complicações vasculares possíveis após um transplante renal incluem trombose da veia e artéria renais, estenose da artéria renal, pseudoaneurisma e dissecção da artéria ilíaca. Entre essas diversas complicações, a estenose da artéria renal transplantada é a mais comum, enquanto a dissecção da artéria ilíaca é a mais rara. Relatamos o caso de um homem idoso que desenvolveu tanto dissecção da artéria ilíaca quanto estenose da artéria renal transplantada 2 meses após transplante renal. As intervenções endovasculares percutâneas foram bem-sucedidas em ambas as complicações. O período pós-intervenção cursou sem complicações, com melhora na função renal do enxerto e na perfusão do membro inferior esquerdo.

## INTRODUCTION

Vascular complications following renal transplantation are common, ranging from 1.3 to 9% in various series.^1^^-^4 These include renal artery and vein thrombosis, renal artery stenosis, pseudoaneurysm, and iliac artery dissection.^1^^-^4 Iliac artery dissection is a rare iatrogenic complication,^4^ which is usually apparent during surgery or in the immediate post-operative period. Transplant renal artery stenosis (TRAS) is another vascular complication, which is relatively common and usually manifests within a few months to a year following the surgery.^1^^-^4 Both complications need a repeat surgical intervention, however, a less invasive percutaneous intervention may be a safe alternative in these cases. We hereby report a case of successful percutaneous intervention for iliac artery dissection and TRAS in a post-renal transplant patient.

## CASE REPORT

**Figure 1:**
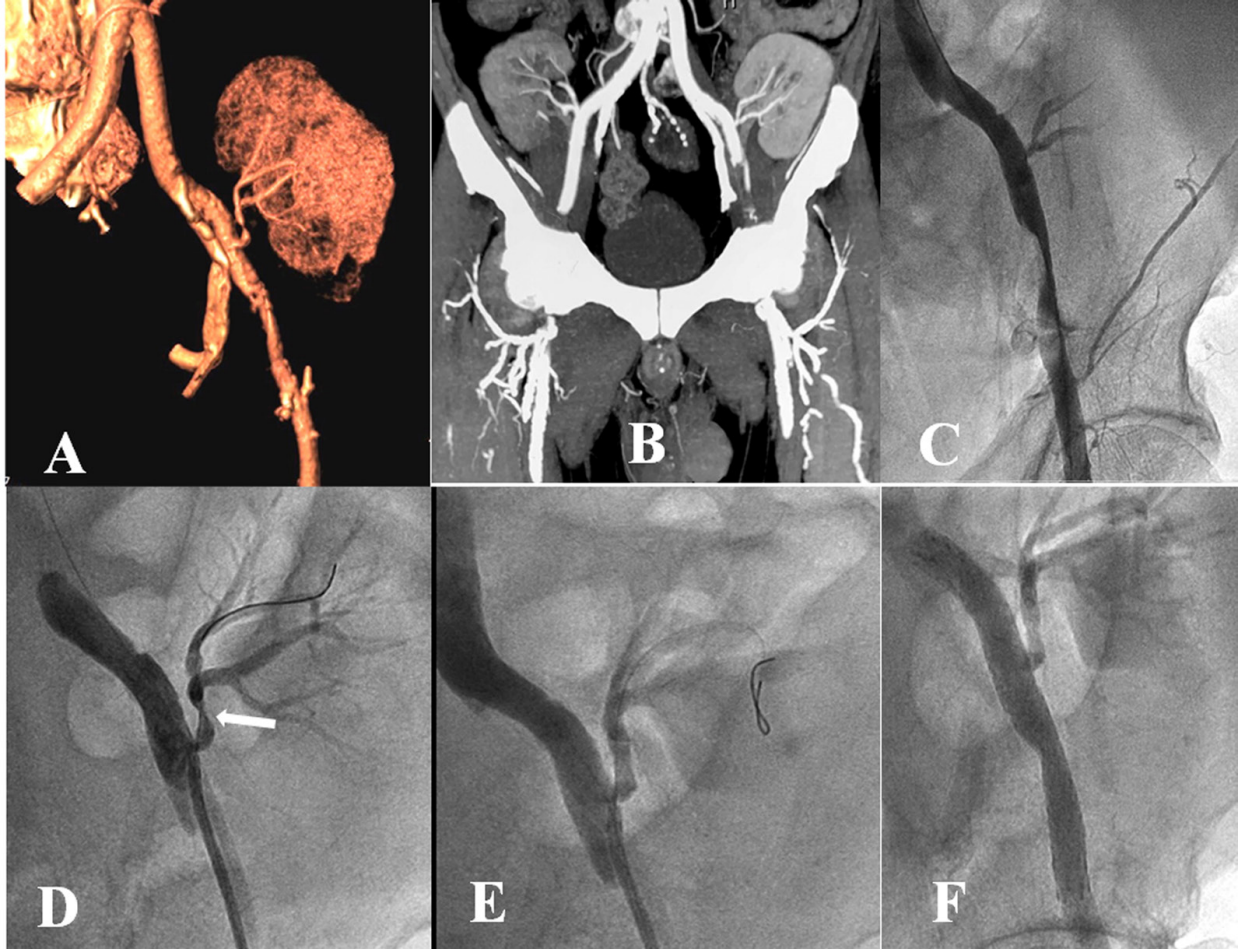
Reconstructed volume-rendered **(A)** computed tomography angiogram with maximum intensity image projection **(B)** showed 90% stenosis of the transplanted renal artery **(A)**, and left external iliac artery (EIA) dissection **(A)** with 90% stenosis **(B)**. Bilateral renal grafts anastomosed to the EIA can be seen **(B)**. The peripheral angiogram showed 90% stenosis with dissection of the left EIA **(C)** and 90% stenosis of the proximal part of the transplanted renal artery **(D, White arrow)**. Brisk flow was achieved across the transplanted renal artery **(E)** and left EIA **(F)** following endovascular intervention.

A 60-year-old male with end-stage renal disease had repeat renal allograft transplantation following loss of a 5-year-old renal allograft, secondary to chronic rejection. Two months following the surgery, he presented with a progressive rise in serum creatinine from 1.0 to 1.9 mg/dL. On examination, his left femoral artery was feeble, and the left lower limb ankle-brachial index was 0.6. Doppler ultrasound of the graft renal artery showed increased peak systolic velocity (300 cm/s) and resistive index (0.8) suggestive of renal artery stenosis. Computed tomography angiogram showed 90% luminal stenosis of the graft renal artery (Figure 1A). Additionally, there was a dissection of the left external iliac artery (EIA) with 90% luminal narrowing (Figure 1A and 1B). The old nonfunctional renal graft could be seen on the right side (Figure 1B). He was referred from the renal transplant surgery department to our unit for endovascular intervention.

Arterial access via the left femoral artery was taken. A contrast angiogram confirmed the left EIA dissection with luminal narrowing (Figure 1C) and left TRAS (Figure 1D). The EIA was dilated with a 7 X 60 mm balloon. The stenosed graft renal artery was dilated with a 4 X 15 mm balloon, following which there was brisk flow across it with no significant residual stenosis (Figure 1E). Later, the left EIA was stented with a 9 X 80 mm self-expanding stent (*Absolute Pro, Abbott Vascular, Santa Clara, CA, USA*). The final angiogram showed brisk flow across both EIA and graft renal artery (Figure 1F). Following the intervention, his left lower limb ankle-brachial index was 0.98. Serum creatinine gradually decreased to 1.2 mg/dL in the next 2 weeks. He had an asymptomatic 1 year of follow-up. Repeat ultrasound at last follow-up revealed peak systolic velocity of 180cm/s, across the transplanted renal artery, normal color flow, and intrarenal resistive index of 0.7. The ultrasound images were not stored and a repeat examination was not feasible due to the prevailing COVID-19 pandemic and patient unwillingness to visit the hospital again.

Informed written consent was taken for the percutaneous intervention of arterial disease. The case report is in accordance with the Helsinki Convention and was approved by the institutional ethics committee for retrospective evaluation.

## DISCUSSION

Vascular complications remain a common concern following renal transplant.^1^^-^4 TRAS is the most common vascular complication,^1^^-^4 while iliac artery dissection is a rare complication.^4^ TRAS can present early within 3 months to late up to 2 years following transplantation.^1^^,^^2^^,^^5^

The site of TRAS can be the donor renal artery, at the suture site, or the recipient artery.^2^^,^^4^^,^^5^ Etiology includes surgical suture technique, damage to vessel endothelium during graft harvesting or surgery, atheroma of the donor artery, external mechanical compression, and, rarely, immune-mediated injury.^2^^,^^4^^,^^5^ We had TRAS at the anastomotic suture site within 2 months of surgery, which was possibly due to defective suturing or injury at the site. Most reported EIA dissections were observed during the early postoperative period.^4^^,^^6^ Iatrogenic injury/dissection usually occurs during anastomosis of the graft kidney and vascular clamping of the EIA. The index case had raised creatinine following ischemic insult of the graft kidney secondary to TRAS and EIA dissection. The iatrogenic dissection remained undiagnosed for the initial 2 months following surgery. Later, with the onset of renal dysfunction and on evaluation of possible vascular complications, both TRAS and EIA dissection were diagnosed. Early detection and management of these vascular complications are important to reduce morbidity and graft loss.^1^^-^5 There are earlier reports of open surgery for TRAS management.^2^^,^^4^^,^^7^ However, percutaneous renal angioplasty has become the treatment of choice in the current era.^1^^,^^5^^,^^8^^,^^9^ There are limited published case reports about surgical^4^^,^^6^^,^^10^^,^^11^ and endovascular^12^^,^^13^ management of EIA dissection following renal transplantation.

Management of such a patient, having both of these vascular complications, poses a challenge, especially when the other site for repeat graft anastomosis is lost. Surgical management in such a situation would require localized endarterectomy with patch/graft repair of TRAS^2^^,^^4^^,^^7^ and polytetrafluoroethylene (PTFE) graft interposition to repair the EIA, along with replantation of transplant renal artery to grafted EIA.^4^^,^^6^^,^^10^^,^^11^ Such extensive vascular surgery would have a high complication rate including loss of renal graft, re-exploration, lower limb ischemia, and mortality.^2^^,^^7^^,^^10^ Endovascular treatment as chosen in the index case was a less invasive, safe, and effective option for this complex vascular complication. Following the endovascular intervention, we were able to normalize the renal function of the graft kidney and perfusion of the left lower limb. To the best of our knowledge, there are no published case reports of simultaneous endovascular intervention for EIA dissection and TRAS in a patient following renal transplantation. In conclusion, we hereby describe an elderly male who had ischemic renal graft dysfunction following TRAS and EIA dissection, which were successfully managed with endovascular treatment.

## References

[1] Srivastava A, Kumar J, Sharma S, Abhishek, Ansari MS, Kapoor R (2013). Vascular complication in live related renal transplant: an experience of 1945 cases. Indian J Urol.

[2] Dimitroulis D, Bokos J, Zavos G (2009). Vascular complications in renal transplantation: a single-center experience in 1367 renal transplantations and review of the literature. Transplant Proc.

[3] Salehipour M, Salahi H, Jalaeian H (2009). Vascular complications following 1500 consecutive living and cadaveric donor renal transplantations: a single center study. Saudi J Kidney Dis Transpl.

[4] Ayvazoglu Soy EH, Akdur A, Kirnap M, Boyvat F, Moray G, Haberal M (2017). Vascular Complications After Renal Transplant: A Single-Center Experience. Exp Clin Transplant.

[5] Hurst FP, Abbott KC, Neff RT (2009). Incidence, predictors and outcomes of transplant renal artery stenosis after kidney transplantation: analysis of USRDS. Am J Nephrol.

[6] Tsai S-F, Chen C-H, Hsieh S-R, Shu K-H, Ho H-C (2013). Salvage of external iliac artery dissection immediately after renal transplant. Exp Clin Transplant..

[7] Benoit G, Moukarzel M, Hiesse C, Verdelli G, Charpentier B, Fries D (1990). Transplant renal artery stenosis: experience and comparative results between surgery and angioplasty. Transpl Int.

[8] Seratnahaei A, Shah A, Bodiwala K, Mukherjee D (2011). Management of transplant renal artery stenosis. Angiology.

[9] Marini M, Fernandez-Rivera C, Cao I (2011). Treatment of transplant renal artery stenosis by percutaneous transluminal angioplasty and/or stenting: study in 63 patients in a single institution. Transplant Proc.

[10] Merkus JW, Dun GC, Reinaerts HH, Huysmans FT (1992). Iliac artery dissection after renal transplantation. Nephrol Dial Transplant.

[11] Dar TI, Tyagi V, Khawaja AR, Chadha S, Jauhari H (2016). External iliac artery polytetrafluoroethylene graft interposition: An effective rescuer for kidney transplant in progressive intimal dissection of external iliac artery. Urol Ann.

[12] Kimura T, Saito T, Tsuchiya T (2005). Treatment of external iliac artery dissection with endovascular stent placement in a patient with simultaneous pancreas and kidney transplantation. Transplant Proc.

[13] Vijayvergiya R, Sharma A, Singh S, Kanabar K (2019). Endovascular treatment of an external iliac artery dissection causing early renal graft dysfunction. Ann Vasc Surg.

